# Polymer Film Blend of Polyvinyl Alcohol, Trichloroethylene and Cresol Red for Gamma Radiation Dosimetry

**DOI:** 10.3390/polym13111866

**Published:** 2021-06-04

**Authors:** Aris Doyan, Susilawati Susilawati, Saiful Prayogi, Muhammad Roil Bilad, Muhamad Fatikul Arif, Noor Maizura Ismail

**Affiliations:** 1 Science Education Program, University of Mataram, Jl. Majapahit No. 62, Mataram 83125, Indonesia; 2 Physics Education, FKIP, University of Mataram, Jl. Majapahit No. 62, Mataram 83125, Indonesia; 3 Faculty of Applied Science and Enginering, Universitas Pendidikan Mandalika UNDIKMA, Jl. Pemuda No. 59A, Mataram 83126, Indonesia; saifulprayogi@ikipmataram.ac.id (S.P.); muhammadroilbilad@ikipmataram.ac.id (M.R.B.); 4 Department of Materials Engineering, Institut Teknologi Sumatera, Lampung Selatan 35365, Indonesia; mf.arif@mt.itera.ac.id; 5 Faculty of Engineering, Universiti Malaysia Sabah, Jln UMS, Kota Kinabalu 88400, Malaysia; maizura@ums.edu.my

**Keywords:** optical properties, polymer film composite, γ-rays irradiation, dosimetry

## Abstract

This study investigated the polymer film composite of polyvinyl alcohol (PVA), trichlorethylene (TCE) and cresol red (CR) dye irradiated with gamma (γ) rays for potential application as radiation dosimetry. The film was prepared via the solvent-casting method with varying concentrations of TCE. Film samples were exposed to radiation from a γ-rays radiation source of ^60^Cobalt isotope. Color changes before and after γ-rays irradiation were observed, and the optical properties of the polymer films were investigated by spectrophotometry. Results show that increasing the radiation dose physically changed the color of the polymer film, from purple (pH > 8.8) without radiation (0 kGy) to yellow (almost transparent) (2.8 < pH < 7.2) at the highest dose (12 kGy). The concentration of acid formed due to irradiation increased with the increase in irradiation doses and at higher TCE content. The critical doses of PVA-TCE composites decreased linearly with the increase of TCE composition, facilitating an easy calibration process. The dose response at 438 nm increased exponentially with increasing radiation dose, but showed an opposite trend at the 575 nm band. An increase in the TCA concentration indicated a decrease in the absorption edge and an increase in activation energy, but both decreased for all TCE concentrations at higher doses. The energy gap for the direct and the indirect transitions decreased with increasing TCE concentration and γ-rays radiation dose. The results of this study demonstrated the potential application of PVA-TCE-CR polymer film as γ-rays irradiation dosimetry in a useful dose range of 0–12 kGy.

## 1. Introduction

Dosimeters from various materials have been intensively studied as devices to monitor radiation doses [1]. Dosimeters of colored thin-film polymer materials have been extensively developed for measuring the adsorbed radiation dose by materials, and have been applied in routine dosimeters [2]. The main technical advantage of a polymer film-based dosimeter as a radiation detector is its slightness and portability [3]. In addition, the film has a long storage stability, is sturdy, and is cost-effective [4]. Some of the applications of film dosimeters include: routine high-dose radiation to food and beverages [5]; sterilization process [6]; radiotherapy in medical field [7]; and dye dosimeters [8,9,10].

Radiation dosimetry is used to measure the absorbed radiation dose, or determine the incident radiation on a material [11]. Therefore, it is necessary to ensure the accuracy of the radiation dose [12,13]. Many materials have been developed and explored as radiation dosimetry, evaluated under different dose ranges [14,15,16]. A film-based radiation dosimetry can be developed from a mixture of polymers, with a dye as indicator. The polymer materials that have been explored include polyvinyl alcohol (PVA) [5,13]; polycarbonate [17]; polyvinyl chloride [18]; and polyvinyl butyric [14]. Meanwhile, coloring materials as indicators include: methylene blue and methylene red [4]; thymolphthalein (TP) [5]; ethyl violet and blue bromophenol [19]; cresol red (CR) [20]; tetrazolium violet [21]; and methyl viologen [22].

PVA based polymer materials are most recommended because they have a high degree of flexibility [23], are water-soluble [24], have good mechanical properties, and are non-toxic and elastic [25]. PVA has been combined with several mixed dyes (tetrabromo phenolphthalein ethyl ester, acid yellow, and chloral hydrate) and has shown promising prospect as a new dosimeter in the 0.1 to 5 kGy dose range [1]. PVA with TP dye is effective as a new detector system for application at doses of 1 to 6.5 kGy [5], and PVA with methyl thymol blue dye showed some efficacy under a dose range from 2.5 to 20 kGy [11].

Several blends of chlorine-containing polymer have been investigated for possible use for dosimetry of γ-rays radiation and electron beams [26,27]. They also contain dye as pH indicators, and the presence of chlorine improves the water solubility. For instance, a mixture of dehydrochlorines and an acid has a low pH. The low pH increases the sensitivity of the dye component to change color.

In this study, we explored the potential of PVA blended with trichlorethylene (TCE) and CR dye as a radiation dosimetry. The addition of a TCE that contains chlorine is expected to enhance the solubility and stabilize the pH [28], as well as increase the dye sensitivity [29]. TCE is also found to be an electro-catalyst in polymers [30]. In order to be applied as a radiation dosimetry, the optical properties of the PVA-TCE-CR polymer film need to be further explored. In this study, we investigated the optical characteristics of the PVA-TCE polymer film with CR dye, and irradiated with γ-rays at doses of 0 to 12 kGy. Several samples with TCE variations (20%, 25%, 30%, and 35%) were fabricated and characterized.

## 2. Materials and Methods

### 2.1. Polymer Film Preparation

Polymer film composites were prepared from the following components: PVA, TCE, CR dye, and color thinners (ethanol and NaOH). The film from the mixture was prepared using the solvent-casting method [31]. A stock dye solution of the polymer film was prepared by mixing CR 0.08 g (SDS for 105225, Merck, Darmstadt, Germany) with 50 mL of ethanol (96% technical, Merck, Darmstadt, Germany), and 10% NaOH (Merck, Darmstadt, Germany). The mixture was then stirred for 10 min at room temperature until homogeneous. The prepared CR dye solution was placed in a closed container (bottle) at a room temperature of 25 °C until further use.

The polymer film was prepared by dissolving 17.5 g PVA (Mw = 72,000 g/mol, Sigma-Aldrich, St Louis, MO, USA) with 350 mL distilled water in a beaker. This mixture was heated at 80 °C while stirring using a magnetic stirrer at 150 RPM in an open container (to allow evaporation) for 4 h until the remaining volume of solution was 50 mL. In this condition, TCE (Mw = 131.39 g/mol, Sigma-Aldrich, St Louis, MO, USA) was added to the mixture while stirring for 1 h. The concentrations of TCE were varied at 20%, 25%, 30%, and 35%. Afterward, the temperature was lowered to 25 °C, then the mixture was added to the stock CR dye solution. The mixture was then continuously stirred for about 20 min until homogeneous.

The homogeneous PVA-TCE-CR solution was poured onto a glass plate and spread evenly to form a thin film. The cast film was then left to stand for the drying process for 120 h at a room temperature of 25 °C. Under this condition, a solid polymer film was formed by a mixture of PVA-TCE-CR. After solidification, the polymer film was cut into a size of 2 cm × 2 cm and stored in a special container ampoule to protect it from dirt and sun exposure, at room temperature. The average thickness of the resulting polymer films was 75 ± 1 μm, measured using a digital micrometer (Mitutoyo Corporation, Kanagawa, Japan).

### 2.2. Polymer Film Irradiation

The polymer film was irradiated with γ-rays (Gamma Irradiator ISG-500), sourced from ^60^Co pencil types (C-188 Cobalt-60 Sources, Nordion, Ottawa, ON, Canada) with an activity of 2 × 250 kCi and an average γ-energy of 1.25 MeV. The decay of a ^60^Co nucleus releases one electron with 317.9 keV energy and two γ quanta with energies of 1.173 MeV and 1.332 MeV. The films were irradiated with 1.25 MeV γ-rays from a J. L Sherpered type γ-ray 60Co source at a mean dose rate of 163.75 Gy.min^−1^. As such, the irradiation dosing rates were adjusted by manipulating the irradiation time. A total of 48 polymer film samples of four TCE concentrations (20%, 25%, 30%, and 35%) were irradiated under different doses of 1 to 12 kGy at room temperature. As benchmarks, four samples of the polymer film were not irradiated (0 kGy) for each TCE concentration. The measurements were taken five times for each condition without any significant variations and presented as averages. The physical changes in the color of the film with or without irradiation were compared. From trial and error in the preliminary experiments, it was found that a small step of less than 1 kGy was insignificant in changing the color of the films. A step of 1 kGy was found significant and thus applied in the experiments. Moreover, these kinds of films are aimed to be used for sterilization applications that require up to 12 kGy.

### 2.3. Optical Properties Analysis

Measurement of the optical absorption of polymer films under all radiation doses and concentrations was done using a UV-Vis spectrophotometer (UV-1900i, WL range: 190–1100 nm, WA: +/− 0.1-nm, Shimadzu, Canby, OR, USA). The scanning was done over a wavelength range of 300 to 700 nm. The optical absorption characteristics were plotted in the form of a graph to show wavelength vs. absorbance relationships. Measurements were made on each film sample that had been irradiated by γ-rays with four variations of TCA concentrations (20%, 25%, 30%, and 35%). The formation of acid in film composites, critical dose at color change, optical absorption dose response, absorption edge (*A_E_*), activation energy (Δ*E*), and energy gap (*Eg*) were then evaluated. The absorption edge and activation energy were determined according to the Urbach edges method [32], and the optical energy gap was determined according to the Mott and Davis model [33].

## 3. Results and Discussion

### 3.1. Discoloration of the Polymer Film before and after Radiation

**Figure 1:**
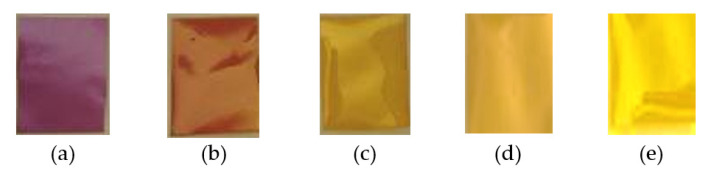
Appearance of the polymer film sample with 20% TCE after radiation with doses of (**a**) 0 kGy; (**b**) 2 kGy; (**c**) 4 kGy; (**d**) 6 kGy; and (**e**) 12 kGy.

The color of the PVA-TCE-CR polymer film samples before and after γ-rays irradiation experienced significant changes as shown in Figure 1. Increasing the dose of γ-rays irradiation physically changed the color of the polymer film samples, from purple (pH > 8.8) without radiation (0 kGy) to yellow (leading to transparency) (2.8 < pH < 7.2) at the highest dose (12 kGy). These findings show that exposure to γ-rays energy at different doses changed the color of the film, in which the dose played an important effect. The change of color was consistent for all variations of TCE concentrations. The decrease of the sample pH was caused by the presence of acids resulting from the interaction of γ-rays with water molecules and TCE.

The change in color can be ascribed to the decrease in the sample’s pH, caused by the presence of acid generated from the interaction of γ-rays with water molecules and TCE, a chlorine-containing substance. There was no color change for the dyed PVA films prepared without TCE added (for one concentration), even though it was irradiated to 12 kGy. This suggests that only TCE molecules of the PVA-TCE composites were affected by γ-rays irradiation within the applied dose range. Another study reported that the polymer film of PVA-chloral hydrate-TPBE-AY dyes irradiated by γ-rays produced colors from green to yellow to red, due to a decrease in pH that occurred due to HCl produced from chloral radiolysis [1].

The impact of irradiation on color changes found in this study is consistent with previous reports for different polymer film components. Previous studies showed that the blue color intensity of the polymer film mixture of methyl thymol blue and PVA decreased gradually with the increase in the γ-rays radiation dose. The color transition was attributed to the formation of a large number of free radicals due to radiation exposure, which gradually increased the rate of blue color reduction in the polymer film samples [11]. The γ-rays interactions produced hydrated electrons and free radicals that damage the dye material molecules and remove chromophores [34,35]. Increasing the radiation dose also led to a gradual bleaching of the polymer samples, as reported elsewhere [36]. In another report, the chlorine bonding of the mixed film polymer was dehydrochlorinated due to γ-rays irradiation, which increased the chlorine ion in the film [29].

### 3.2. Absorption Spectra

**Figure 2:**
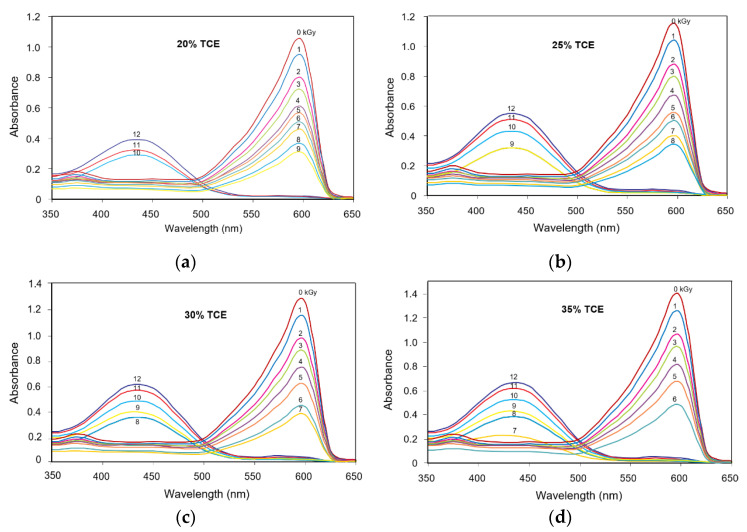
Absorbance spectra of CR dyed PVA-TCE composites containing; (**a**) 20%; (**b**) 25%; (**c**) 30%; and (**d**) 35% TCE irradiated with γ-rays at various doses.

Figure 2 shows the absorption spectrum of the polymer blend film with TCE composition at 20%, 25%, 30% and 35%. The absorption spectrum of the PVA-TCE-CR composites were measured before and after γ-irradiation with variable doses (0 to 12 kGy). Two absorption peaks at 438 and 575 nm bands were found to be consistent for all tested samples. A band at 575 nm served as the main absorbance peak of the purple color characteristic of the PVA-TCE-CR polymer film composite. At 20% TCE, films with a radiation dose of 0 kGy (unirradiated) to the one irradiated with 9 kGy maintained the main absorption peak at 575 nm. However, the main absorbance peak of polymer films irradiated with 10, 11 and 12 kGy shifted from 575 nm to 438 nm. The peak shifting for TCE concentration of 25%, 30% and 35% occurred for irradiation doses of 9, 10, 11 and 12; 8, 9, 10, 11 and 12; 7, 8, 9, 10, 11 and 12 kGy, respectively.

The absorption spectra of the unirradiated films show a main absorption peak at the 575 nm band (a characteristic of observed purple color). Upon irradiation, the absorbance at the 575 nm band decreased gradually, while at the absorption peak of the 438 nm band (a characteristic of observed yellow color) emerged with increasing intensity at higher doses.

These results are consistent with the results of previous studies on composite polymer film PVA-trichloroacetid acid (PVA-TCA) which produced the same absorption bands (575 nm and 438 nm) as expected for most organic compounds containing chlorine [29]. However, they have a different dose response. For a given absorption dose, the absorbance of PVA-TCE composites in the 575 nm band was higher than that of the PVA-TCA composites, but for the 438 nm band, it was higher for the PVA-TCA composites than for the PVA-TCE composites. This difference within the literature data may be due to various factors, such as sample thickness, dose sensitivity, and the concentration of acid formed in the two sample systems.

The colorimetric property associated with the change in the optical absorption peak due to gamma radiation on a film is an important aspect in radiation dosimetry. In this study, we identified highly visible results within a 1 to 12 kGy dose range that enable the polymer film materials to be used in many dosimetry applications using ^60^Co. At low doses (<5 kGy), the film can be used as a dosimetry label or indicator for food irradiation processing, medical product sterilization, and polymer modification [1], while for high doses (>6 kGy), it can be applied to various control processes in industrial radiation facilities [13].

### 3.3. Formation of Acid in PVA-TCE Composites

**Figure 3:**
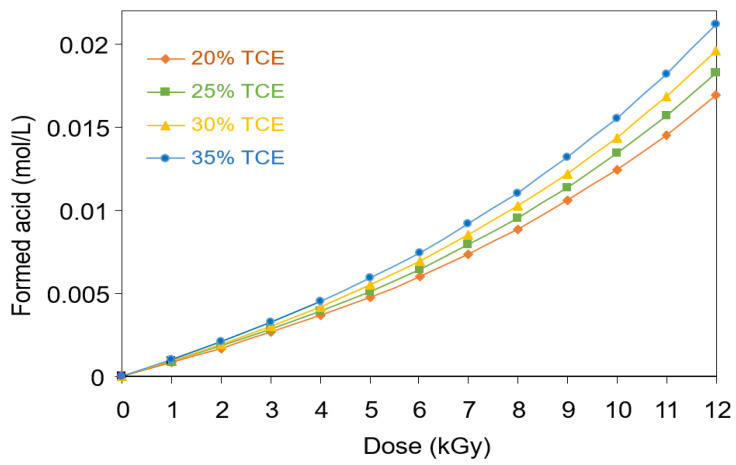
Concentration of acid formed as function of dose in PVA-TCE-CR polymer films with different compositions of TCE derived from the absorbance at 438 nm.

Figure 3 shows the concentration of acid formed in the PVA-TCE samples containing different TCE compositions as a function of the absorbed dose. It could be seen that the concentration of acid formed increased with the increase in the irradiation dose and the TCE content. Upon irradiation, the TCE in the polymer film was dechlorinated, in which chlorine ions detached from the carbon backbone of TCE. Thus, the excited TCE dissociated to radicals, which may be represented by Equation (1).

(1) $$C_{2}{\mathrm{HCl}}_{3}*\;\to \;\dot{C_{2}{\mathrm{HCl}}_{2}}+\dot{Cl}$$

The radicals of hydrogen H* and hydroxyl OH* from hydrolysis of water, and Cl* from TCE recombined to form other chemical products including hydrochloric acid.

Figure 3 shows the concentration of acid formed in the PVA-TCE-CR polymer film composites during irradiation with γ-rays. It can be seen that the acid concentration was dependent on the dose and the composition and type of blend added. The acid formed increased with increasing TCE concentration from 20% to 35% and with the radiation dose up to 12 kGy.

Similar finding on acidification of irradiated polymer film were also reported in earlier studies. For a single carbon bond (C–C) containing compound in PVA-chloral hydrate composite, the acid concentration at dose 12 kGy reached 1.0 mol L^−1^ for 34% CH [37], while at the same radiation dose reached of 0.18 mol L^−1^ for 35% TCA (in PVA-composite) [29]. However, the acid formed in a double carbon bond (C=C) containing compound such as PVA-TCE composites, the acid formed was much smaller at 0.015 mol L^−1^ for 20% TCE and 0.022 mol L^−1^ for 35% TCE when irradiated at the dose 12 kGy. The increasing trend can be attributed to more energy of the photon required to break a covalent bond involving a C=C compound. It follows that the amount of acid formed and the subsequent chemical and physical effects of irradiated PVA composites was influenced by the type of carbon bond of the compounds.

### 3.4. Critical Dose at Color Change

**Figure 4:**
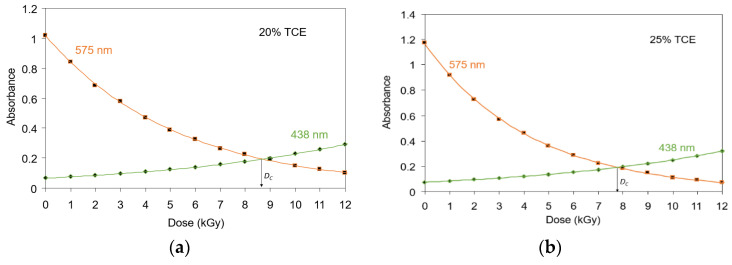

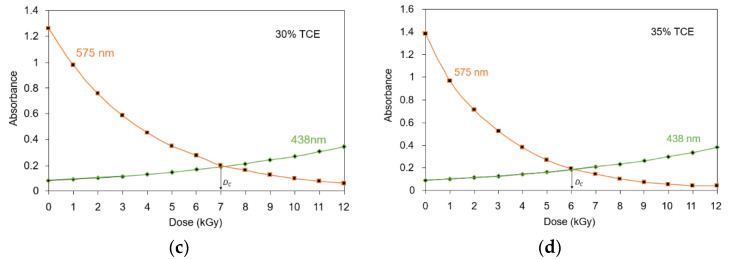
Critical doses determined as the intersection of absorbance at 575 nm and 438 nm bands for PVA-TCE-CR polymer film containing (**a**) 20%, (**b**) 25%, (**c**) 30%, and (**d**) 35% TCE.

The shift of the main absorption bands from 575 nm to 438 nm as the result of γ-rays irradiation on the film occurred at a certain dose point, called a critical dose, and has been shown in Figure 2. In Figure 4, the critical dose of each tested TCE concentration is presented by evaluating the intersection of absorption curves at 438 nm and 575 nm bands for each TCE composition. The dose at this intersection was taken as the critical dose (*D_C_*) at which the polymer film changed color from more purple/violet to more yellow (Figure 1). Figure 4 also shows the useful critical dose as a function of TCE composition. The values obtained from 20%, 25%, 30%, and 35% of TCE were ranged at 8 to 9 kGy, 7 to 8 kGy, 7 kGy, and 6 kGy, respectively.

**Figure 5:**
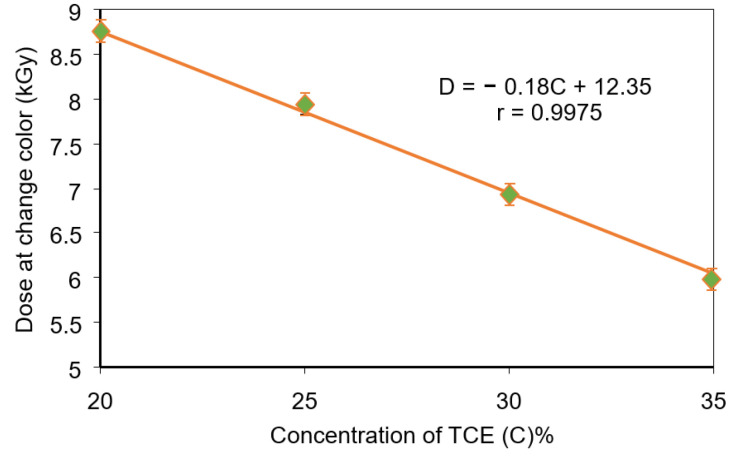
Useful critical doses as a function of TCE concentration for PVA-TCE-CR polymer films.

Figure 5 shows critical doses as a function of TCE composition for PVA-TCE-CR polymer film. The critical dose decreased linearly with the increase of TCE composition and has a relationship given by *D_C_* = −0.18*C* + 12.35 (r = 0.99), where *C* is the composition of TCE. It shows that he critical dose of polymer film composites decreased linearly with increasing TCE compositions.

### 3.5. Optical Absorption Dose Response

**Figure 6:**
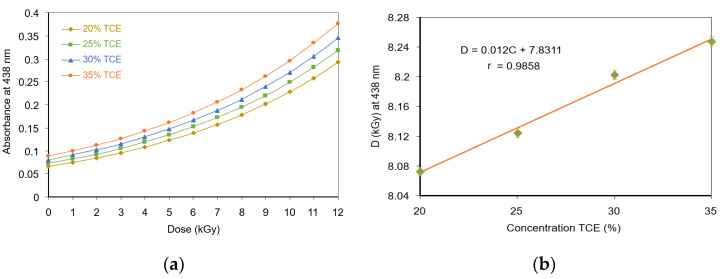

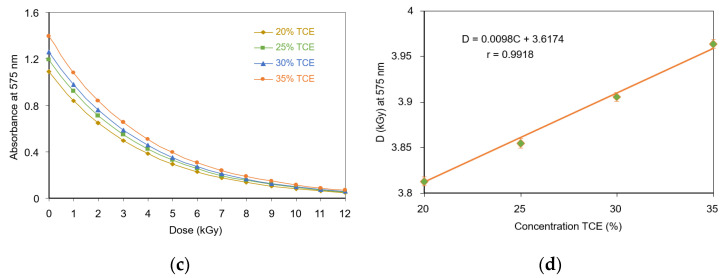
Optical absorption dose response; (**a**) dose response curve at 438 nm band; (**b**) sensitive dose *D*_0_ vs. TCE composition as derived from 438 nm band; (**c**) dose response curve at 575 nm band; (**d**) sensitive dose *D*_0_ as a function of TCE composition as derived from 575 nm band.

The radiation dose response for each absorption band was evaluated as a function of the TCE content in the PVA-TCE-CR polymer films. The dose-response curves at 438 nm increased exponentially with doses as shown in Figure 6a. The data fitted well with a mathematical model of $y=y_{0}e^{D/D_{0}}$. The dose sensitivity parameter *D*_0_ obtained had a function of *D*_0_ = 0.012*C* + 7.8311, where *C* is the composition of TCE, as shown in Figure 6b.

For the dose-response curves at 575-nm band, which decreased exponentially with dose, a mathematical model of $y=y_{0}e^{-D/D_{0}}$ was used (Figure 6c). The results show that *D*_0_ had the relationship of *D*_0_ = 0.0098C + 3.6174, where *C* is the composition of TCE, as shown in Figure 6d. Since, *D*_0_ showed a linear relationship with the TCE composition, the dose response of the film is thus desirable for ease of calibration and interpretation as a radiation dosimetry.

### 3.6. Absorption Edge

**Figure 7:**
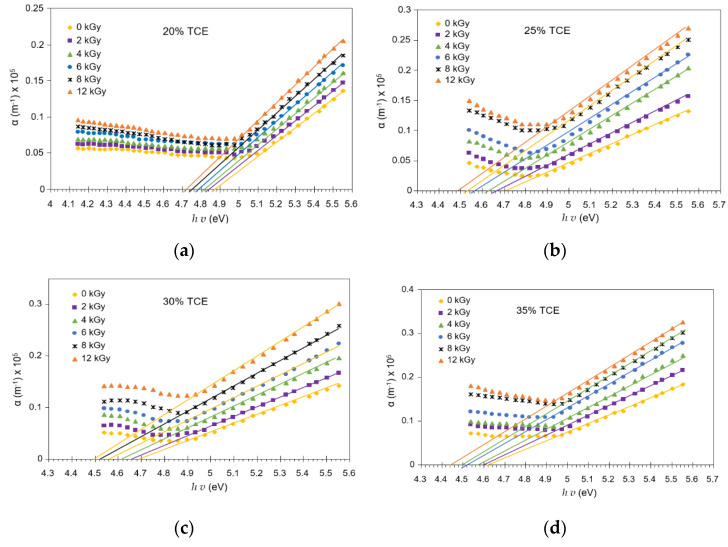
Relationship between α(*v*) vs. *hv* under different doses for; (**a**) 20%; (**b**) 25%; (**c**) 30%; and (**d**) 35% of TCE content in PVA-TCE-CR polymer film.

The absorption of UV spectra increased with the increasing dose, as shown in Figure 7. This band corresponded to the excitation of outer electrons attributed to the π−π* electronic transitions of electrons from donor atoms (HOMO) to acceptor atoms (LUMO) of the film. The absorption coefficient, *α*(*v*), of dyed PVA-TCE film was determined from the optical absorption spectrum. The plots of *α*(*v*) vs. *hv* at different doses are shown in Figure 7 for different TCE compositions. Near the absorption edge, α increased more rapidly with *hv*. The absorption edge was determined by extrapolating the linear portions of α(*v*) vs. *hv* curves to zero value of the absorption coefficient.

**Figure 8:**
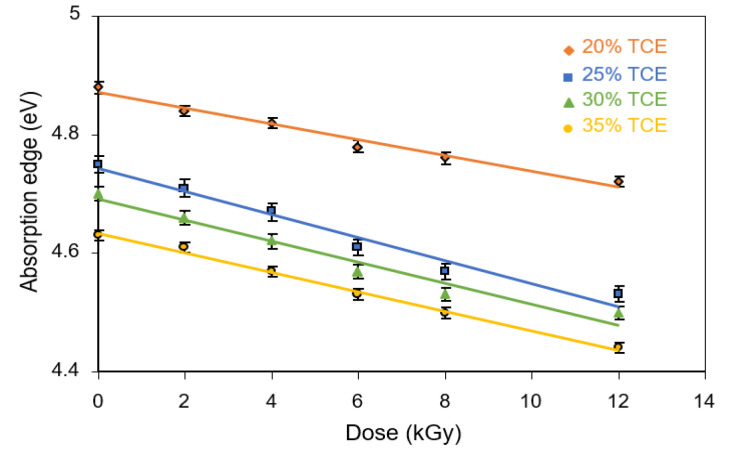
Absorption edge of PVA-TCE-CR polymer films as a function of dose for different TCE compositions.

The absorption edge decreased with increasing TCE composition and increasing dose as shown in Figure 8. The absorption edge of dyed PVA-TCE film decreased for 20% TCE from 4.88 to 4.72 eV when the dose increased from 0 to 12 kGy. For the same radiation condition, it decreases from 4.63 to 4.44 eV for 35% TCE. When compared with literature data, at about the same blend composition, the absorption edge of the PVA-CH film was higher than that the PVA-TCE film [37], followed by the PVA-TCA film [29]. Overall, the absorption edge of irradiated PVA-TCE composites was higher than that of the UPVC (4.35 to 2.04 eV) [38].

For pure PVA film, the absorption edge was found to be around 5.34 eV [39]. In the present study, this value was reduced by 0.9 eV, under 35% PVA-TCE and a dose of 12 kGy to about 4.44 eV. A greater trend of decreasing absorption edge with increasing radiation dose was also found in polymer films blended with salts, such as PVA-AgNO_3_ polymer film irradiated with γ-rays at high doses. At doses of 20 to 50 kGy, it produced an absorption edge of 1.43. to 0.96 eV [40].

### 3.7. Activation Energy

**Figure 9:**
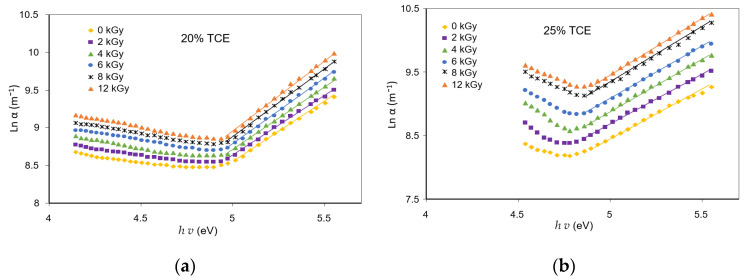

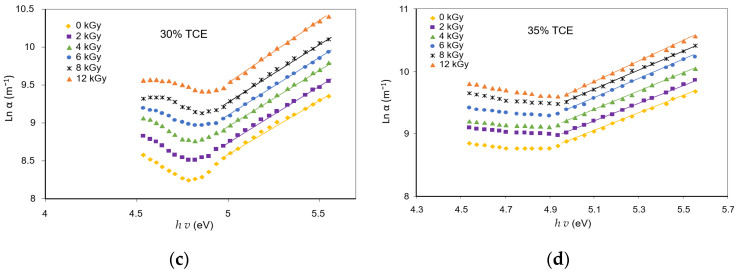
Variation of ln(*α*) vs. h*ν* at various doses for; (**a**) 20%; (**b**) 25%; (**c**) 30%; and (**d**) 35% TCE composition of CR dyed PVA-TCE composites.

The optical activation energy was evaluated using the Urbach edges method [32]. The activation energy of irradiated samples was determined from the slope of the straight line of ln(*α*) versus photon energy *hv* for different TCE compositions (Figure 9). The activation energy in a reaction is defined as the amount of energy required to start a reaction. This represents the minimum energy required to form a complex motion in the event of a collision between reagents [41].

**Figure 10:**
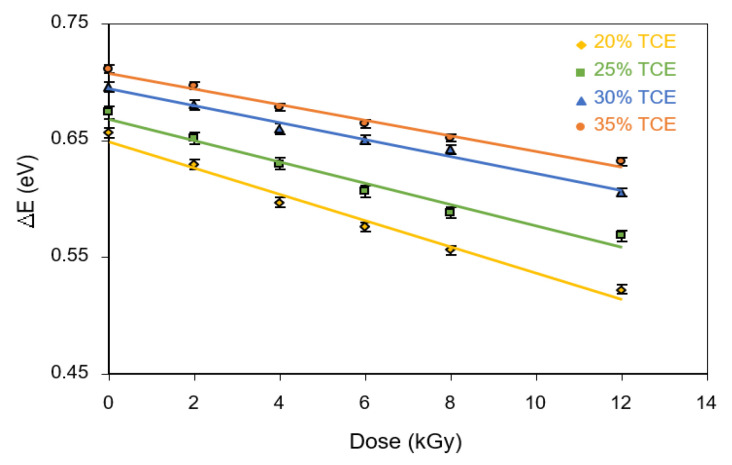
Effect of γ-rays irradiation and TCE composition on the optical activation energy (Δ*E*) of CR dyed PVA-TCE composites.

From the results shown in Figure 10, it can be seen that activation energy decreased with the increase of the radiation dose as well as TCE concentration. It was found that the activation energy value at 0 Gy increased from 0.66 eV for the 20% TCE to 0.72 eV for the 35% TCE. At 12 kGy, the value increases from 0.49 eV for the 20% TCE to 0.65 eV for the 35% TCE. Therefore, activation energy increased with the increase of the TCE composition and decreased at higher doses. These findings are supported by the results of previous studies, that the activation energy decreased with an increasing dose of γ-rays irradiation [29] as a result of chain-scission polymeric molecules in polymer samples [42]. Evaluation of previous studies revealed that activation energy values of PVA-TCE composites were higher than that of PVA-TCA composites for all tested compositions and doses [29]. This finding suggests that radiation dose affected significantly the change of the width of the tail of the localized states of the energy band.

### 3.8. Band Gap Energy

**Figure 11:**
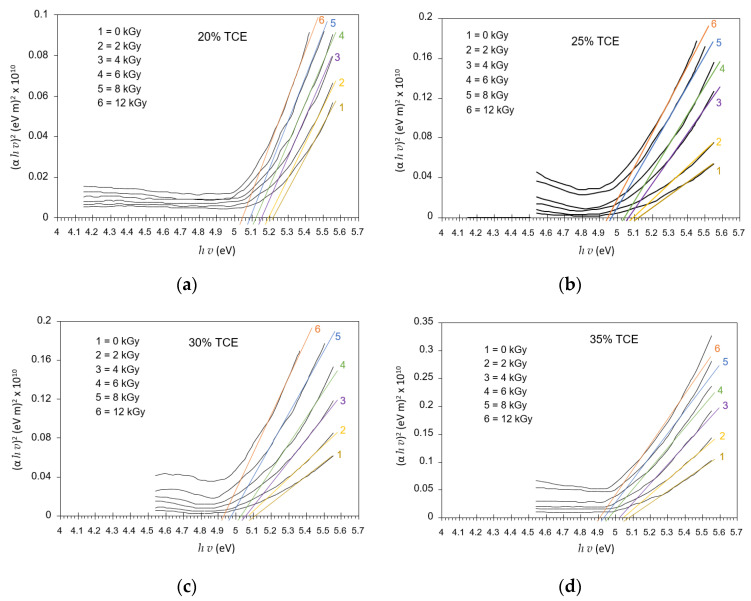
Variation of direct allowed transition (*αhv*)^2^ vs. *hv* at various doses for PVA-TCE-CR polymer film at; (**a**) 20%; (**b**) 25%; (**c**) 30%; and (**d**) 35% TCE composition.

Figure 11 shows extrapolation (*α*h*ν*)^m^ versus hν that resulted in a variation energy gap for each radiation dose and concentration. The energy gap was determined according to the Mott and Davis model [33]. Band gap energy or energy gap is the energy range in the absence of electrons from a material; it lies between the valence and conduction bands [43]. Enough energy is required to make the transition of these two bands [44]. Optical absorption spectrum analysis can be used to determine the optical energy gap between the valence band and the conduction band due to direct and indirect transitions [45,46]. The direct optical band gaps in UV region were evaluated from (*αhv*)^2^ versus *hv* at different doses, as illustrated in Figure 11.

**Figure 12:**
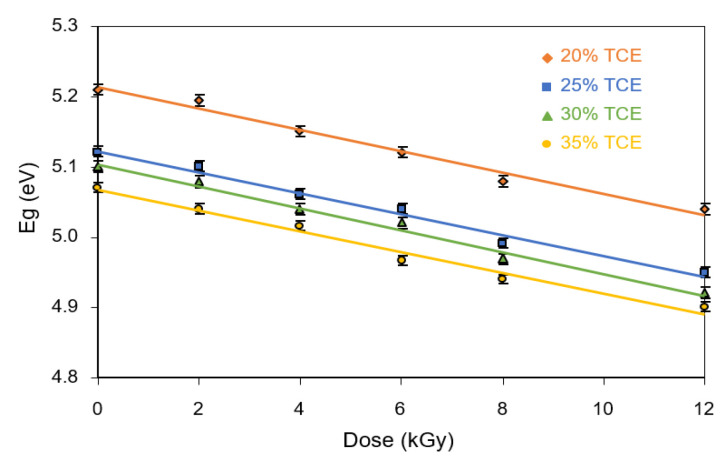
Variation of the direct energy band gaps with dose for PVA-TCE-CT polymer films at different TCE compositions.

Figure 12 summarizes the relationship between the optical band gaps and dose at different TCE compositions. The results show that the direct energy gap decreased with increasing doses for all TCE compositions. It was found that the energy gap at 0 Gy decreased from 5.21 eV for the 20% TCE to 5.07 eV for the 35% TCE. At 12 kGy, the value decreased from 5.04 eV for the 20% TCE to 4.90 eV for the 35% TCE. The energy gap value of the PVA-TCE film was slightly smaller than that of the PVA-TCA films under all doses [29].

**Figure 13:**
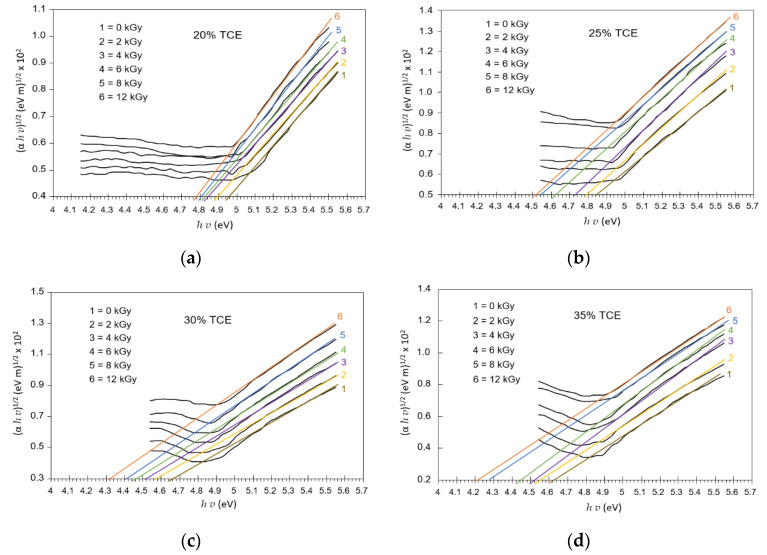
Variation of indirect allowed transition (*αhv*)^1/2^ vs. *hv* at various doses PVA-TCE-CT polymer films at; (**a**) 20%; (**b**) 25%; (**c**) 30%; and (**d**) 35% TCE composition.

**Figure 14:**
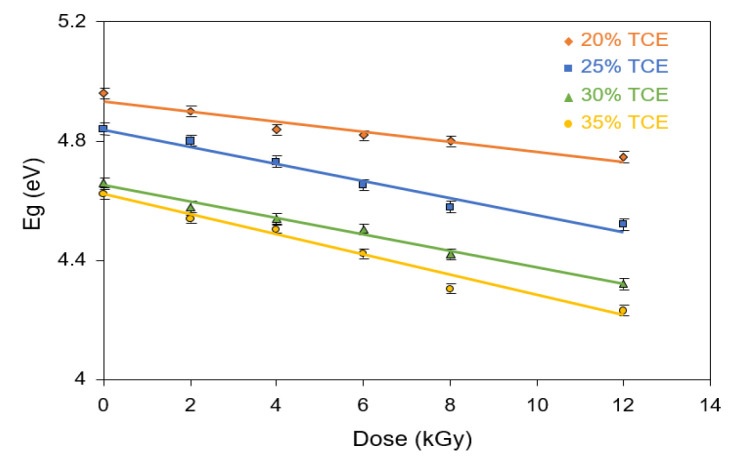
Variation of the indirect energy band gaps with dose for CR dyed PVA-TCE films at different TCE compositions.

The indirect optical band gap energy of the UV region was evaluated from the linear plots of (*αhv*)^1/2^ vs. *hv* under different doses, as illustrated in Figure 13. The extrapolation, for which (*αhv*)^1/2^ = 0 yielded the indirect optical band gap, was a function of the dose, as illustrated in Figure 14. The indirect band gap decreased with the increase in dose for all TCE compositions. It had similar features to that of the direct band gap, but the value of the indirect band gap energy was always smaller. It was found that at 0 Gy, the indirect energy gap decreased from 4.96 eV for the 20% TCE to 4.62 eV for the 35% TCE. At 12 kGy, the value decreased from 4.74 eV for the 20% TCE to 4.23 eV for the 35% TCE. It was found also that the indirect band gaps of the PVA-TCE composites were larger than that of the PVA-TCA composites [29], for the same compositions and doses. The decrease in the band gap resulted from the increase in polarons and free ions in the polymer sample, due to exposure to the γ-rays irradiation, as explained elsewhere [44,47].

Overall, these results suggest that the amount of energy gap in the irradiated polymer material depends on the type and composition of the dopants under the influence of γ-ray irradiation. The response of the optical properties of the material to the radiation dose is very important to be investigated. The results of previous studies showed a linear response of decreasing energy gaps to γ-rays irradiation on TeO_2_ thin films observed in the dose range of 0 to 37 Gy, which resulted in an energy gap in the range of 3.75 to 345 eV [48]. The linear response decreased the energy gap to 4.16 and 4.34 eV for KCl-Mn and KCl-Ce phosphorus polymer materials irradiated with γ-rays at doses of 0.08 to 0.75 kGy [49].

The behavior of the optical properties of the studied material specimens under the influence of radiation dose is important to identify its potential application in radiation dosimetry systems. They usually show varying responses to the dose exposed to the material, such as linear, supralinear, saturated response, and defective with increasing radiation dose [50]. In this present work, the energy gap for both types of transitions (direct and indirect) showed a linear decreasing response to radiation dose. Linearity indicated that the material has stable optical properties which can be used as a promising dosimetry [48,49].

## 4. Conclusions

The PVA-TCE-CR polymer film composite has been introduced for γ-rays irradiation dosimetry applications. The study of its optical properties was explored before and after γ-rays irradiation. Results showed that increasing the radiation dose physically changed the color of the polymer film, from purple (pH > 8.8) without radiation (0 kGy) to yellow (almost transparent) (2.8 < pH < 7.2) at the highest dose (12 kGy), demonstrating its effective use as dosimetry. The concentration of acid formed increased at a higher dosing rate and composition of TCE, which affected the color transition of the irradiated films. The critical doses of film composites decreased linearly with the increase of TCE compositions. The dose response at 438 nm increased exponentially with increasing radiation doses. Conversely, the dose response at the 575 nm band decreased with increasing radiation doses. An increase in the TCA concentration indicated a decrease in the absorption edge and an increase in activation energy, but both decreased for all TCE concentrations at higher doses. The energy gap for the direct and the indirect transitions decreased with increasing TCE concentration and γ-rays radiation dose. The results of this study indicated the potential application of PVA-TCE-CR polymer film as γ-rays irradiation dosimetry in a useful dose range of 0–12 kGy. We have identified highly visible results within a 1 to 12 kGy dose range, allowing the PVA-TCE-CR based polymer film composite to be applied in many dosimetry applications using ^60^CO. At doses of <5 kGy, it is applicable as a dosimetry label or indicator for food irradiation processing and polymer modification, while for doses of >6 kGy, it can be applied to medical product sterilization and various control processes in radiation facilities.

## Data Availability

The data presented in this study are available on request from the corresponding author.
